# Risk factors of ICU-acquired weakness in sepsis patients

**DOI:** 10.12669/pjms.41.9.12558

**Published:** 2025-09

**Authors:** Changyu Jin, Fulin Hu, Ling Wu, Liuhui Zhang, Bo Wu, Yupei Yang

**Affiliations:** 1 Changyu Jin Department of Emergency Intensive Care Unit, First Affiliated Hospital of Anhui Medical University, 218 Jixi Road, Hefei, Anhui Province 230022, P.R. China; 2 Fulin Hu First Affiliated Hospital of Anhui Medical University, 218 Jixi Road, Hefei, Anhui Province 230022, P.R. China; 3 Ling Wu Department of Emergency Intensive Care Unit, First Affiliated Hospital of Anhui Medical University, 218 Jixi Road, Hefei, Anhui Province 230022, P.R. China; 4 Liuhui Zhang Department of Emergency Transfusion Room, First Affiliated Hospital of Anhui Medical University, 218 Jixi Road, Hefei, Anhui Province 230022, P.R. China; 5 Bo Wu Dept. of Anesthesiology, Anhui University of Chinese Medicine, 117 Meishan Road, Hefei, Anhui Province 230031, P.R. China; 6 Yupei Yang Department of Emergency Intensive Care Unit, First Affiliated Hospital of Anhui Medical University, 218 Jixi Road, Hefei, Anhui Province 230022, P.R. China

**Keywords:** Acquired weakness, Intensive care units, Risk factors, Sepsis

## Abstract

**Background & Objective:**

Sepsis patients in intensive care units (ICUs) are at high risk of ICU-acquired weakness (ICU-AW). This study aimed to analyze the risk factors of ICU-AW in sepsis patients.

**Methodology:**

This retrospective cohort study included clinical data of 149 sepsis patients hospitalized in the ICU of the First Affiliated Hospital of Anhui Medical University from January, 2022 to December, 2024. Based on the incidence of acquired weakness (AW), patients were divided into the ICU-AW and non-ICU-AW groups. Risk factors were assessed through univariate logistic regression analysis and adjusted for odds ratio (OR) using binary logistic regression analysis. The Receiver Operating Characteristic (ROC) curve was generated and the predictive value of each risk factor for ICU-AW occurrence was determined.

**Results:**

A total of 65 patients (43.6%) developed ICU-AW. Univariate analysis showed significant differences between the two groups in terms of gender, ICU length of stay, protective restraint, mechanical ventilation time, initiation time of nutritional support, nutritional support method, used sedatives and long-term bed rest > 7 days (P<0.05). Multivariate regression analysis showed that gender (OR=0.060; 95% CI: 0.007-0.535) and ICU length of stay (OR=9.728; 95% CI: 3.693-25.623) were independent influencing factors of ICU-AW (*P*<0.05). The ROC curve analysis showed that the length of ICU stay and the combined prediction had high predictive value for ICU-AW. The area under the curve (AUC) of combined prediction (0.987; 95% CI: 0.974-0.999) was higher than that of individual indicators (0.981; 95% CI: 0.965-0.997).

**Conclusions:**

In the management of sepsis patients in the ICU, special attention should be paid to the risk of weakness in male patients and long-term hospitalized patients and targeted preventive measures should be taken.

## INTRODUCTION

Sepsis is characterized by a deregulated systemic response to infection, frequently leading to multiple organ dysfunction. It is considered one of the leading causes of mortality within the intensive care unit (ICU), often manifesting in polyneuropathy, diffuse muscle weakness, muscle atrophy and limb paralysis.^1^-^3^

Current research reports a correlation of sepsis with the occurrence of the ICU-Acquired Weakness (ICU-AW), a syndrome of unexplained muscle weakness and functional impairment in ICU patients.^3^-^7^ The most common risk factors for ICU-AW include sepsis, duration of mechanical ventilation, acute physiology and chronic health assessment II scores, blood lactate levels, length of ICU stay and the use of sedatives.^5^-^7^ A study by Zhang et al.^8^ also identified female gender, old age, body mass index (BMI), Simplified Acute Physiology Score (SAPS) II score, Acute Physiology and Chronic Health Evaluation (APACHE-II) II score, hyperglycemia, duration of mechanical ventilation and use of neuromuscular blockers at ICU admission as predictive factors for ICU-AW. However, due to the variability in the results of different studies, the pathogenesis of ICU-AW and the predictive value of the identified risk factors in the onset of ICU-AW are still unclear.

This retrospective analysis aimed to determine the independent influencing factors of ICU-AW and evaluate their predictive value. Therefore, the aim of this study was to identify independent risk factors for ICU-acquired weakness in sepsis patients admitted to the ICU and to evaluate the predictive performance of these factors.

## METHODS

This retrospective cohort study was conducted to determine independent predictors of ICU-AW in ICU sepsis patients and assess the discriminative performance of these factors using logistic regression and ROC curve analysis. This retrospective cohort study collected clinical data of 149 sepsis patients treated in the ICU of the First Affiliated Hospital of Anhui Medical University from January 2022 to December 2024. Based on the occurrence of ICU-AW, patients were divided into the ICU-AW group (n=65) and the non-ICU-AW group (n=84). Based on the Medical Research Council (MRC) score, ICU-AW was diagnosed when a patient scored less than 48 points for 12 muscle groups in the limbs at intervals of 24 hours or more, with each muscle group scoring less than four points.^9^ MRC scoring was performed by two trained ICU physicians independently. Evaluations were repeated after at least 24 hours to confirm ICU-AW diagnosis. Due to the retrospective nature of the study, the assessments were not blinded but were based on routine clinical documentation.

### Ethics approval and consent to participate:

All procedures performed in the study involving human participants were in accordance with the ethical standards of the institutional and/or national research committee(s) and the Helsinki Declaration (as revised in 2013). The requirement for informed consent was waived by the ethics committee of the First Affiliated Hospital of Anhui Medical University due to the observational and retrospective nature of the study. This study was approved (Ref. No.: PJ 2025-02-83; dated May 9, 2025) by the ethics committee of the First Affiliated Hospital of Anhui Medical University.

### Inclusion criteria:

- Meet the diagnostic criteria for sepsis 3.0.^10^
- Age ≥ 18 years old.
- ICU length of stay ≥ 72 hours.

### Exclusion criteria:

- Pre-existing neuromuscular disorders before admission.
- Diseases that can cause neuromuscular dysfunction, such as traumatic brain injury, spinal cord injury, cerebrovascular disease, brain tumors, Guillain Barr é syndrome, etc.
- Continuous coma, paraplegia, limb joint surgery, fractures, lower limb thrombosis, etc. must be stopped.
- ICU hospitalization history within six months.
- Pregnant or lactating women.
- Patients with incomplete clinical data.

### Collected indexes:

The collected data included age, gender, BMI, APACHE-II score, ICU length of stay, hypertension, diabetes, heart disease, kidney disease, septic shock, multiple organ dysfunction syndrome (MODS), smoking history, drinking history, protective restraint, mechanical ventilation time, continuous renal replacement therapy (CRRT), tracheal intubation, tracheotomy, procalcitonin (PCT), blood glucose, C-reactive protein (CRP), lactate dehydrogenase (LDH), lactate (Lac), hemoglobin (Hb), creatinine (Cre), albumin (Alb), platelet (PLT), start time of nutrition support, nutrition support mode, application of vasoactive drugs, used sedative, glucocorticoids, neuromuscular blockers and long-term bed rest braking > 7 days. Bed rest >7 days was defined as continuous bedridden status without active limb movement or ambulation for more than seven days. Passive range-of-motion exercises administered by healthcare providers were not considered interruption of bed rest.

### Statistical analysis:

SPSS 22.0 (IBM Corp, Armonk, NY, USA) was used as the statistical software. The normality of the distribution of continuous variables was evaluated using the Shapiro-Wilk test. Data that followed a normal distribution (such as age, APACHE-II score, etc.) were represented as mean ± standard deviation and an independent sample t-test was used to compare the two groups. The data that did not follow a normal distribution (such as PCT, blood glucose levels, etc.) were represented by M(P25/P75). The Mann-Whitney *U* test was used to compare the two groups. Count data (such as gender and distribution of hypertension) were represented as n (%) and intergroup comparisons were conducted using chi-square tests. Variables with statistical significance in univariate analysis were included in multivariate logistic regression analysis to determine independent risk factors of ICU-AW. A multicollinearity test was performed on factors with P<0.05. If the tolerance was greater than 0.05 and the variance inflation factor (VIF) was less than 10, a lack of multicollinearity was determined and the data were included in the multivariate correlation analysis. Binary logistic regression was used to adjust for the odds ratio (OR). The Hosmer-Lemeshow test model fit was expressed as the adjusted OR with a 95% confidence interval (95% CI). Receiver Operating Characteristic (ROC) curves were generated to evaluate the predictive value of various risk factors for sepsis ICU-AW. Predictive value of each indicator was evaluated through the ROC curve: the area under the curve (AUC) value closer to one indicated better the predictive performance of the model. *P*<0.05 was statistically significant. All clinical data were checked for completeness before analysis. Patients with incomplete data were excluded based on the study’s exclusion criteria. As a result, no missing values were present in the final dataset.

## RESULTS

**Fig.1:**
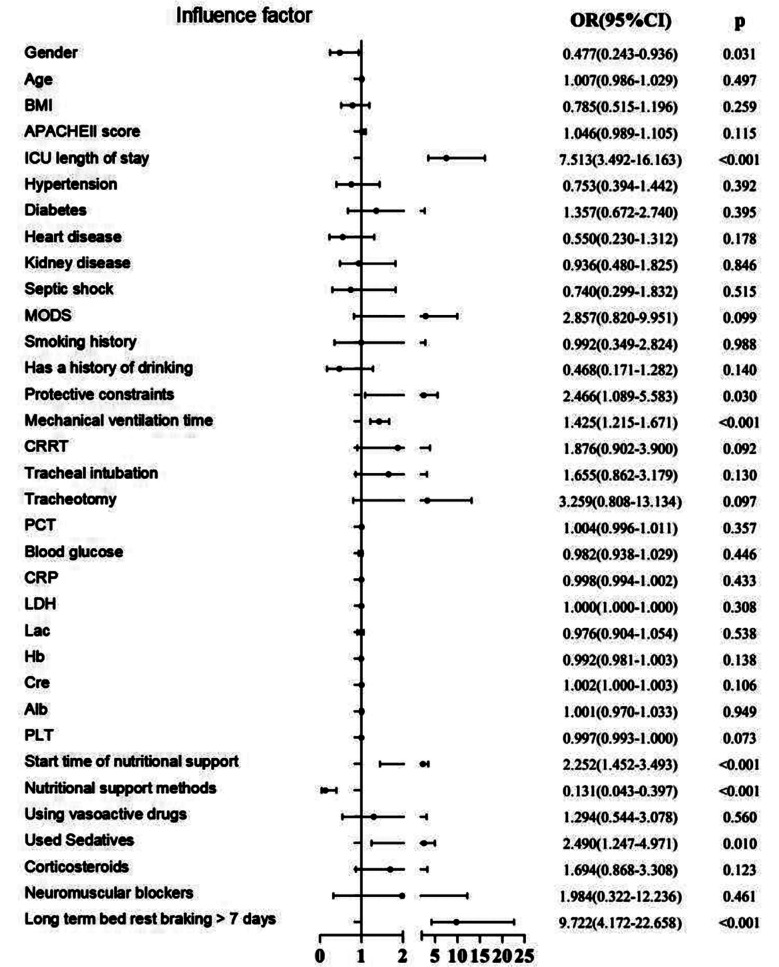
Univariate logistic regression analysis of risk factors affecting ICU-AW in sepsis patients.

This study included and analyzed clinical records of 149 patients (86 males and 63 females) aged 18-96 years, with an average age of 62.3 ± 15.6 years. Among them, 65 patients developed ICU-AW, with a prevalence rate of 43.6% (65/149). The results of univariate analysis showed a significant difference between the ICU-AW and the non-ICU-AW group in terms of gender, ICU length of stay, protective restraint, mechanical ventilation time, initiation time of nutritional support, nutritional support method, used sedatives and long-term bed rest braking > 7 days (all *P*<0.05). Table-I Univariate logistic regression analysis that used the eight potential influencing factors related to ICU-AW as independent variables showed significant differences between the two groups in terms of gender, ICU length of stay, protective restraint, mechanical ventilation time, initiation time of nutritional support, nutritional support method, used sedatives and long-term bed rest > 7 days. Fig.1 The results of the multicollinearity test showed that the tolerances of the included eight factors ranged from 0.270 to 0.989 and the VIF ranged from 1.011 to 3.698, indicating a low probability of multicollinearity (Table-II).

**Table-I:** Comparison of various indicators between two groups.

Indicators	Non-ICU-AW group (n=84)	ICU-AW group (n=65)	χ^2^/t/z	P
Gender (yes), n(%)			4.700	0.030
Male	42(50.00)	44(67.69)		
Female	42(50.00)	21(32.31)		
Age (years), mean±SD	61.6±16.4	63.3±14.5	-0.676	0.500
BMI (kg/m²), n(%)			3.163	0.531
<18.5	2(2.38)	2(3.08)		
18.5-22.9	33(39.29)	28(43.08)		
23-24.9	34(40.48)	28(43.08)		
25-29.9	13(15.48)	7(10.77)		
≥30	2(2.38)	0(0)		
APACHEII score, mean±SD	20.0±5.8	21.6±6.2	-1.591	0.114
ICU length of stay (days), M(P25/P75)	3(2,4)	8(6,11)	-10.124	<0.001
Hypertension (yes), n(%)	46(54.76)	31(47.69)	0.733	0.392
Diabetes (yes), n(%)	23(27.38)	22(33.85)	0.727	0.394
Heart disease (yes), n(%)	19(22.62)	9(13.85)	1.848	0.174
Kidney disease (yes), n(%)	53(63.1)	40(61.54)	0.038	0.846
Septic shock (yes), n(%)	73(86.90)	54(83.08)	0.427	0.514
MODS (yes), n(%)	4(4.76)	8(12.50)	2.919	0.088
Smoking history (yes), n(%)	9(10.84)	7(10.77)	0.000	0.988
Has a history of drinking (yes), n(%)	15(17.86)	6(9.23)	2.252	0.133
Protective constraints (yes), n(%)	58(69.05)	55(84.62)	4.847	0.028
Mechanical ventilation time (days), M(P25/P75)	0(0,1)	2(0,7)	-3.766	<0.001
CRRT (yes), n(%)	18(21.43)	22(33.85)	2.877	0.090
Tracheal intubation (yes), n(%)	36(42.86)	36(55.38)	2.303	0.129
Tracheotomy (yes), n(%)	3(3.57)	7(10.77)	1.992	0.158*
PCT (ng/ml), M(P25/P75)	10.9(2.4,33.6)	9.96(1.92,26.32)	-0.548	0.584
Blood glucose (mmol/L), M(P25/P75)	7.3(5.4,12.25)	7.5(5.08,10.2)	-0.530	0.596
CRP (mg/L), M(P25/P75)	133.34(63.11,190.55)	114.67(72.63,162.57)	-0.480	0.631
LDH (U/L), M(P25/P75)	286(212.5,401)	324(240,489)	-1.523	0.128
Lac (mmol/L), M(P25/P75)	2.47(1.7,5.5)	2.72(1.65,4.5)	-0.285	0.775
Hb (g/L), M(P25/P75)	108.5(92,125.5)	99(83,119)	-1.910	0.056
Cre (μmol/L), M(P25/P75)	143.9(83.3,238.8)	135.2(70,305.4)	-0.308	0.758
Alb (g/L), M(P25/P75)	29.7(26.35,33.05)	29.1(23.9,32.6)	-1.070	0.285
PLT (× 10^9^/L), M(P25/P75)	91(43,175)	64(35,144)	-1.638	0.101
Start time of nutritional support (days), M(P25/P75)	1(1,2)	2(1,3)	-3.287	0.001
Nutritional support methods, n (%)			16.052	<0.001
Enteral area	0(0)	0(0)		
Extraintestinal + Intraintestinal	56(66.67)	61(93.85)		
Whole exointestinal area	28(33.33)	4(6.15)		
Using vasoactive drugs (yes), n(%)	68(80.95)	55(84.62)	0.341	0.559
Used sedatives (yes), n(%)	43(51.19)	47(72.31)	6.832	0.009
Corticosteroids (yes), n(%)	45(53.57)	43(66.15)	2.399	0.121
Neuromuscular blockers (yes), n(%)	2(2.38)	3(4.62)	0.086	0.770*
Long term bed rest braking > 7 days (yes), n(%)	9(10.71)	35(53.85)	32.759	<0.001

***Note:***

* Fisher’s Exact Test. SD, standard deviation; CRRT, Continuous renal replacement therapy; PCT, procalcitonin; CRP, C-reactive protein; LDH, lactate dehydrogenase; Lac, lactate; Hb, hemoglobin; Cre, creatinine; Alb, albumin; PLT, platelet.

**Table-II:** Collinearity diagnosis.

Indicators	Collinearity statistics	
	Allow	VIF
Gender	0.989	1.011
ICU length of stay	0.270	3.698
Protective constraints	0.957	1.045
Mechanical ventilation time	0.311	3.215
Start time for nutritional support	0.826	1.211
Nutritional support methods	0.826	1.211
Used sedatives	0.792	1.262
Long term bed rest braking>7 days	0.621	1.609

**Fig.2:**
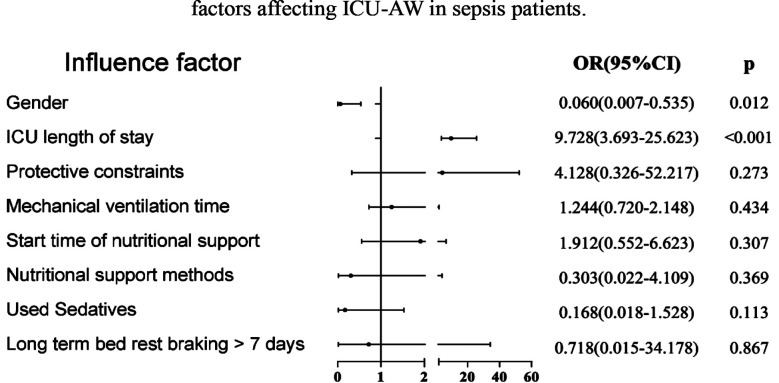
Multivariate logistic regression analysis of independent risk factors affecting ICU-AW in sepsis patients.

Binary logistic regression analysis demonstrated that gender and ICU length of stay were independent influencing factors of ICU-AW (all P<0.05). The results of the analysis showed that compared to males, females were less likely to develop ICU-AW. Additionally, longer ICU stay was associated with increased incidence of ICU-AW (Fig.2).

**Fig.3:**
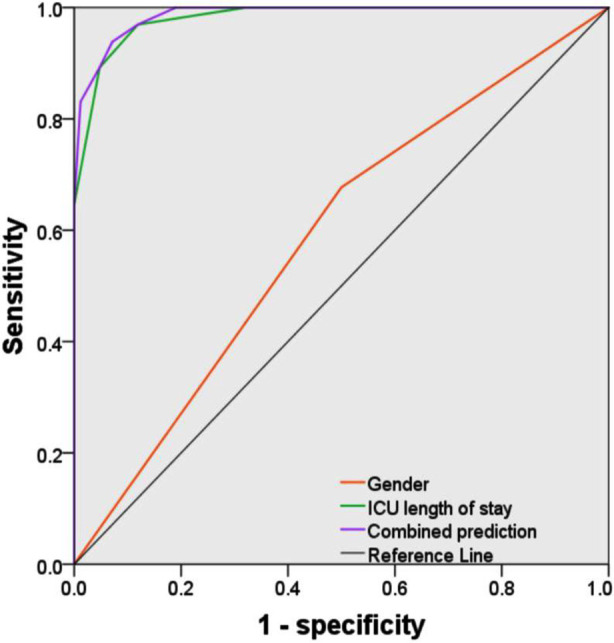
ROC curve of the predictive value of gender, ICU length of stay and combined prediction for ICU-AW in sepsis patients.

The ROC curve analysis showed that gender had no significant predictive value for ICU-AW. In contrast, the length of ICU stay had a high predictive value for ICU-AW (AUC: 0.981; 95% CI: 0.965-0.997). However, gender combined with length of ICU stay has a higher predictive value for ICU-AW (AUC: 0.987; 95% CI: 0.974-0.999) (Table-III) than each factor alone (Fig.3).

**Table-III:** The predictive value of gender, ICU length of stay and combined prediction for ICU-AW.

	AUC	95%CI	P	Sensitivity (%)	Specificity (%)
Female	0.588	0.497-0.680	0.064	67.7	50.0
ICU length of stay	0.981	0.965-0.997	<0.001	96.9	88.1
Joint prediction	0.987	0.974-0.999	<0.001	93.8	92.9

## DISCUSSION

This study identified gender and length of ICU stay as independent risk factors for ICU-AW in septic patients. Male gender and longer ICU stays were significantly associated with a higher risk of developing ICU-AW.

The prevalence of ICU-AW in the current study was 43.6% (65/149), which is similar to the rates reported in previous studies. Liu et al.^11^ analyzed 264 septic patients and demonstrated an ICU-AW prevalence rate of 43.2% (114/264). Similarly, Chen et al.^12^ found that ICU-AW incidence in septic patients exceeded 50%.

However, the pathogenesis of ICU-AW is still unclear. Animal models have confirmed that sepsis inhibits the ability of cells to synthesize myofibrils and sarcoplasmic proteins, leading to metabolic disorders.^13^,^14^ Numerous studies have showed that septic patients often present with organ failure, have malignant tumors, are bedridden for an extended time and are administered sedatives and corticosteroids, which increases the incidence of ICU-AW.^11^,^12^,^15^ It is plausible, therefore, that a higher rate of ICU-AW in these patients results from the possible interaction of multiple risk factors.^7^,^8^,^11^,^12^,^15^

The logistic regression analysis results of this study showed that male and ICU length of stay were independent risk factors of ICU-AW. The role of gender as an independent influencing factor for the occurrence of ICU-AW is still unclear.^16^-^19^ A study by Yang et al.^16^ found that women are at higher risk for ICU-AW. The study by De et al.^18^ also demonstrated a 4.66-fold increase in ICU-AW in female patients compared to males. In contrast, the logistic regression analysis by Wang et al.^19^ showed that gender did not correlate with the occurrence of ICU-AW. The results of this study identified male gender as an independent risk factor for ICU-AW. This discrepancy between outcomes may be due to gender-related hormonal differences, different muscle composition and disease severity, as well as variability in the study population, disease types and diagnostic criteria. Several biological mechanisms may underline the association between male gender and ICU-AW. Elevated androgen levels in males may promote muscle catabolism, while estrogen may confer muscle-protective and anti-inflammatory effects.^18^,^19^ Moreover, sex-related differences in immune responses, with males exhibiting stronger pro-inflammatory reactions, may further contribute to neuromuscular impairment during sepsis.^20^

The study also identified the length of ICU stay as an independent risk factor for ICU-AW, consistent with the previous findings. The median length of ICU stay in the ICU-AW group was 8 (6-11) days, significantly higher than the 3 (2-4) days in the non-ICU-AW group. For every additional day of ICU stay, the risk of ICU-AW increased by 9.728 times (OR=9.728). This result is consistent with the research findings of Yang et al.^17^ and Wang et al.^20^ However, some studies have suggested that the length of ICU stay may be a result rather than a cause of ICU-AW, as muscle weakness lead to weaning failure and prolonged ICU stay.^9^,^21^ This bidirectional relationship suggests that more in-depth research is needed to clarify the causality.

Long-term ICU hospitalization often means more severe illness, more complications and long-term immobilization, all of which may exacerbate muscle atrophy and functional decline.^9^,^21^,^22^ It is worth noting that some factors that showed significant differences in univariate analysis, such as duration of mechanical ventilation and the use of sedatives, did not become independent risk factors in multivariate analysis, which differs from previous studies. Such differences may stem from variabilities in research design, sample size, statistical methods, etc. Some well-established clinical factors such as CRRT use and blood glucose levels did not show significant associations in our study. This may be attributed to the limited sample size, the relatively homogeneous cohort characteristics, and the presence of unmeasured confounders such as glycemic control strategies or renal dysfunction severity. Future larger and multicenter studies are needed to validate these findings. Although the timing and mode of nutritional support were not independent predictors in the multivariate model, their significant association with ICU-AW in univariate analysis suggests potential clinical implications. Early enteral nutrition may reduce inflammation and support muscle preservation, while excessive reliance on parenteral nutrition may be associated with adverse outcomes.^21^ These factors deserve attention in ICU management protocols. In addition, it is important to note that while this study focused exclusively on septic patients, other studies may have included a broader population of ICU patients, which may also lead to differences in results.

The ROC curve analysis showed that gender had no significant predictive value for ICU-AW. In contrast, the length of ICU stay had a high predictive value for ICU-AW (AUC: 0.981; 95% CI: 0.965-0.997). However, gender combined with ICU length of stay demonstrated a superior predictive value for ICU-AW (AUC: 0.987; 95% CI: 0.974-0.999). Although previous studies have suggested possible associations between gender and ICU-AW, our findings add to the current literature by showing that male gender remained an independent risk factor for ICU-AW in a cohort of septic patients. Furthermore, ICU length of stay exhibited a high predictive value (AUC = 0.981), and its combination with gender slightly improved discrimination (AUC = 0.987), indicating potential for a simplified stratification approach. Clinically, these findings highlight the importance of early functional assessment and preventive strategies in male septic patients who experience prolonged ICU stays. Such measures may support muscle preservation, mitigate weakness progression, and enhance post-ICU recovery.

### Study’s strength:

It include the focus on a clearly defined sepsis population, standardized use of the MRC score for ICU-AW diagnosis, and comprehensive inclusion of both clinical and laboratory variables, which strengthen the internal validity and interpretability of the results. Future research should validate these findings in multicenter and more heterogeneous populations. In addition, investigating underlying mechanisms such as hormonal profiles, inflammatory pathways, or neuromuscular electrophysiology—and incorporating objective measurements like electromyography—may further elucidate the biological basis and improve clinical intervention strategies.

### Limitations:

Firstly, the diagnostic basis for ICU-AW is the MRC score, which cannot be evaluated for patients in long-term coma. Moreover, there are subjective differences in MRC scores among different physicians. Secondly, a relatively small sample size may have affected statistical performance. Thirdly, this study was conducted in a single ICU in China, which may limit the generalizability of the findings to other regions or healthcare systems. Future multicenter studies involving diverse populations and settings are necessary to validate these results and enhance external validity. Finally, as the patient’s health condition may improve or deteriorate at any time during their stay in the ICU, the likelihood of developing CU-AW may also change. Future research may consider adopting a multicenter, prospective design, expanding the sample size and combining imaging, electrophysiological and other methods to assess the risk factors for ICU-AW more accurately. Due to the limited sample size, subgroup or interaction analyses were not performed in this study. Future prospective studies with larger sample sizes are warranted to explore potential heterogeneity in risk factor effects.

## CONCLUSION

This study analyzed clinical data of 149 septic patients and found that gender and ICU length of stay were independent risk factors for ICU-AW. Male septic patients are more likely to develop ICU-AW and longer ICU stays are linked to a higher incidence of ICU-AW. Male sepsis patients with ICU stays longer than five days should be considered high-risk for ICU-acquired weakness. Early muscle strength screening and rehabilitation interventions should be implemented to improve outcomes. Future studies are needed to further investigate underlying mechanisms and optimize individualized preventive strategies.

### Authors’ contributions:

**CJ:** Study design, literature search and manuscript writing.

**FH, LW, LZ, BW and YY:** Data collection, data analysis and interpretation. Critical Review.

**YY:** Was involved in the manuscript revision and validation and is responsible for the integrity of the study.

All authors have read and approved the final manuscript.

## References

[1] Liu D, Huang SY, Sun JH, Zhang HC, Cai QL, Gao C (2022). Sepsis-induced immunosuppression:mechanisms, diagnosis and current treatment options. Mil Med Res.

[2] Arshad A, Ahmed W, Rehman N, Naseem Z, Ghos Z (2024). Tackling a deadly global phenomenon:sepsis induced coagulopathy:A narrative review. J Pak Med Assoc.

[3] Ackerman MH, Ahrens T, Kelly J, Pontillo A (2021). Sepsis. Crit Care Nurs Clin North Am.

[4] Angez M, Jassani S, Abbas M, Akbar I, Martins RS, Arshad A (2024). Predictors of clinical outcomes in patients with sepsis:A retrospective study from a tertiary care hospital in Pakistan. J Pak Med Assoc.

[5] Zhou Y, Sun Y, Pan Y, Dai Y, Xiao Y, Yu Y (2025). Risk prediction models for intensive care unit-acquired weakness in critically ill patients:A systematic review. Aust Crit Care Off J Confed Aust Crit Care Nurses.

[6] Piva S, Bertoni M, Gitti N, Rasulo FA, Latronico N (2023). Neurological complications of sepsis. Curr Opin Crit Care.

[7] Yamada K, Kitai T, Iwata K, Nishihara H, Ito T, Yokoyama R (2023). Predictive factors and clinical impact of ICU-acquired weakness on functional disability in mechanically ventilated patients with COVID-19. Heart Lung J Crit Care.

[8] Zhang X, Zang Z, Zhao T, Luo W, Zhang Y, Cao H (2019). Analysis of risk factors for acquired weakness in ICU patients. Chin J Crit Care Med.

[9] Zheng H, Shi Y, Zhang D, Zhao D, Zhao C, Qin B (2024). Research progress of ICU-acquired weakness. Chin Crit Care Med.

[10] Shi QF, Xu Y, Zhang BY, Qu W, Wang SY, Zheng WL (2021). External validation and comparison of two versions of simplified sequential organ failure assessment scores to predict prognosis of septic patients. Int J Clin Pract.

[11] Liu J, Xu Z, Luo S, Bai Y, Feng J, Li F (2024). Risk factors for ICU-acquired weakness in sepsis patients:A retrospective study of 264 patients. Heliyon.

[12] Chen H, Li X, Zhou L, Qiang X (2025). Research progress on ICU-acquired weakness in sepsis patients. Zhonghua Wei Zhong Bing Ji Jiu Yi Xue.

[13] Jiang Y, Wei Q, Liu W, Chen Q, Chen X, Yuan Z (2022). Exploring the Muscle Metabolomics in the Mouse Model of Sepsis-Induced Acquired Weakness. Evid-Based Complement Altern Med ECAM.

[14] Ma Y, Zhang Y, Li R, Deng SW, Qin QS, Zhu LL (2022). Characteristics of amino acid metabolism in myeloid-derived suppressor cells in septic mice. Beijing Da Xue Xue Bao.

[15] She H, Du Y, Du Y, Tan L, Yang S, Luo X (2023). Metabolomics and machine learning approaches for diagnostic and prognostic biomarkers screening in sepsis. BMC Anesthesiol.

[16] Yang Z, Wang X, Chang G, Cao Q, Wang F, Peng Z (2023). Development and validation of an intensive care unit acquired weakness prediction model:A cohort study. Front Med.

[17] Yang Z, Wang X, Wang F, Peng Z, Fan Y (2022). A systematic review and meta-analysis of risk factors for intensive care unit acquired weakness. Medicine (Baltimore).

[18] De Jonghe B, Sharshar T, Lefaucheur JP, Authier FJ, Durand-Zaleski I, Boussarsar M (2002). Paresis acquired in the intensive care unit:a prospective multicenter study. JAMA.

[19] Wang L, Lv H, Shen Y, Jin L, Sheng H (2021). Establishment and validation of a risk prediction model for intensive care unit-acquired weakness. Chin Crit Care Med.

[20] Wang L, Long DY (2024). Significant risk factors for intensive care unit-acquired weakness:A processing strategy based on repeated machine learning. World J Clin Cases.

[21] Sinha RK, Sinha S, Nishant P, Morya AK (2024). Intensive care unit-acquired weakness and mechanical ventilation:A reciprocal relationship. World J Clin Cases.

[22] Ostadi Z, Shadvar K, Sanaie S, Mahmoodpoor A, Saghaleini SH (2019). Thrombocytopenia in the intensive care unit. Pak J Med Sci.

